# Perfluorocarbon reduces cell damage from blast injury by inhibiting signal paths of NF-κB, MAPK and Bcl-2/Bax signaling pathway in A549 cells

**DOI:** 10.1371/journal.pone.0173884

**Published:** 2017-03-21

**Authors:** Zhaorui Zhang, Zhixin Liang, Huaidong Li, Chunsun Li, Zhen Yang, Yanqin Li, Danyang She, Lu Cao, Wenjie Wang, Changlin Liu, Liangan Chen

**Affiliations:** 1 Department of Respiration, Chinese PLA General Hospital, Beijing City, People’s Republic of China; 2 Department of Respiratory Disease, The 88th Hospital of Chinese PLA, Tai’an City, Shandong Province, People’s Republic of China; 3 Department of State Key Laboratory of Explosion Science and Technology, The Beijing University of Technology, Beijing City, People’s Republic of China; Institute of Biochemistry and Biotechnology, TAIWAN

## Abstract

**Background and objective:**

Blast lung injury is a common type of blast injury and has very high mortality. Therefore, research to identify medical therapies for blast injury is important.

Perfluorocarbon (PFC) is used to improve gas exchange in diseased lungs and has anti-inflammatory functions in vitro and in vivo. The aim of this study was to determine whether PFC reduces damage to A549 cells caused by blast injury and to elucidate its possible mechanisms of action.

**Study design and methods:**

A549 alveolar epithelial cells exposed to blast waves were treated with and without PFC. Morphological changes and apoptosis of A549 cells were recorded. PCR and enzyme-linked immunosorbent assay (ELISA) were used to measure the mRNA or protein levels of IL-1β, IL-6 and TNF-α. Malondialdehyde (MDA) levels and superoxide dismutase (SOD) activity levels were detected. Western blot was used to quantify the expression of NF-κB, Bax, Bcl-2, cleaved caspase-3 and MAPK cell signaling proteins.

**Results:**

A549 cells exposed to blast wave shrank, with less cell-cell contact. The morphological change of A549 cells exposed to blast waves were alleviated by PFC. PFC significantly inhibited the apoptosis of A549 cells exposed to blast waves. IL-1β, IL-6 and TNF-α cytokine and mRNA expression levels were significantly inhibited by PFC. PFC significantly increased MDA levels and decreased SOD activity levels. Further studies indicated that NF-κB, Bax, caspase-3, phospho-p38, phosphor-ERK and phosphor-JNK proteins were also suppressed by PFC. The quantity of Bcl-2 protein was increased by PFC.

**Conclusion:**

Our research showed that PFC reduced A549 cell damage caused by blast injury. The potential mechanism may be associated with the following signaling pathways:

1) the signaling pathways of NF-κB and MAPK, which inhibit inflammation and reactive oxygen species (ROS); and 2) the signaling pathways of Bcl-2/Bax and caspase-3, which inhibit apoptosis.

## Introduction

The morbidity and mortality of blast injury are increasing, both on the battleground and in daily life [1]. From 1999 to 2006, a worldwide investigation showed that explosion events have increased four-fold and that injuries caused by explosions have increased eight-fold [2]. Blast injuries are classified into four main categories. A primary blast injury is the direct result of a blast wave. A secondary blast injury is caused by flying debris. A tertiary blast injury is caused by moving bodies and debris pushed by the blast wave. A quaternary injury is the miscellaneous blast injuries caused by the blast, which consist of burns, toxic substance exposure, asphyxia and psychological trauma [3,4]. The air-containing organs, such as the lungs, are at an increased risk of primary blast injury [5,6]. Blast lung injury is the main cause of death in an explosion; approximately 17–47% people die of blast lung injury, and approximately 71% of seriously injured people have pulmonary injury [1,6–9]. However, there are no effective medical therapies for blast lung injury, although medical scientists have tested many methods, such as mechanical ventilation, fluid resuscitation and hyperbaric oxygen [10].

PFC is a colorless and odorless liquid composed of fluorine and carbon. PFC has the following features: high solubility of oxygen and carbon dioxide, fast release of gas, low surface tension, high density, good histocompatibility and deficiency of metabolism and assimilation in vivo [11]. With the “partial liquid ventilation” method, PFC increases and improves lung vital capacity. PFC also increases the ventilation/perfusion ratio in patients with acute respiratory distress syndrome (ARDS) [12,13]. Moreover, animal experiments and clinical trials have shown that PFC decreases the pulmonary inflammatory response in lung injury [14]. Moreover, the levels of cytokines, chemokines and other mediators of pulmonary inflammation are reduced by PFC [14]. Our group found that PFC decreased intercellular adhesion caused by lipopolysaccharide (LPS), which induces damage to A549 cells [15].

Previous studies assessed the mechanism of blast injury using the following different factors: cellular aspect, biochemical aspect and molecular aspect, and the putative biomarkers and targeted therapeutics were similar in vivo and in vitro [16,17]. In our study, we show that PFC attenuates the impact of blast-induced A549 cell injury and suppresses the inflammatory response in vitro.

## Material and methods

### Shock tube and explosion simulation

**Fig 1:**
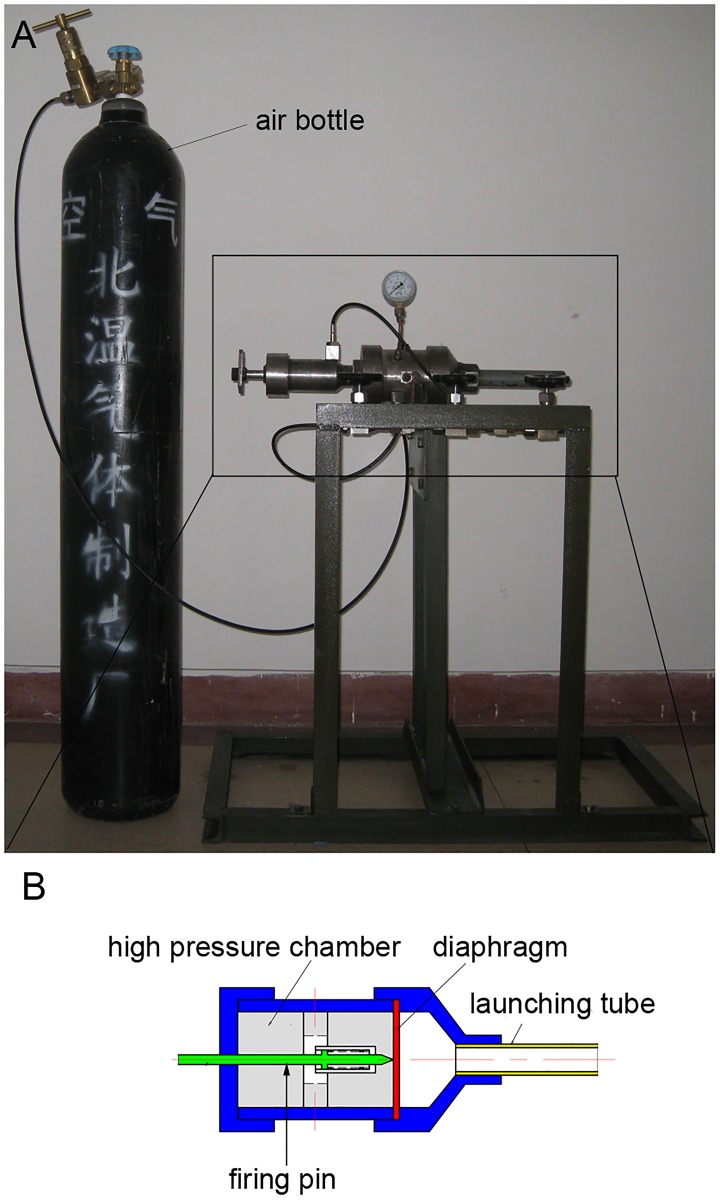
The shock tube. (A) The shock tube and compressed air bottle. (B) Diagram of the shock tube.

The study was conducted from August 2012 to May 2014 in the respiratory laboratory of the Chinese PLA General Hospital. The shock tube is a classical instrument used to replicate direct blast waves to simulate actual explosions and their effects [18,19]. Shock tubes are generally used in biomedical research to study how biological specimens are affected by blast waves [20,21]. The shock tube used in our study was designed by China National Key Laboratory, the Explosion and Technology Lab in Beijing Technology Institute. The shock tube was used to produce peak pressures in the range of 100 to 300 kPa. The actual shock tube is shown in Fig 1a, and a diagram of the shock tube is shown in Fig 1b. The shock tube includes a firing pin, a high-pressure chamber, a diaphragm and a launching tube (Fig 1b). The high-pressure chamber is connected to a compressed air bottle (Fig 1a). The air is transferred from the compressed air bottle to the high-pressure chamber. There is a pressure gauge on the shock tube. When the pressure of the high-pressure chamber reaches 5000 kPa, the firing pin is launched and causes the diaphragm to burst. Then, the compressed air is released from the launching tube and forms a shock wave. The relationship between the distance to the shock tube and pressure detected is shown in Table 1. When the distance between the pressure detector and the shock tube is 60 cm, the pressure detector measured 100 kPa.

**Table 1:** Relationship between pressure and distance.

Distance to the shock tube (cm)	Pressure (kPa)
1.5	300.0
60.0	100.0

### Cell culture

A549 cells (obtained from the respiratory laboratory of the Chinese PLA General Hospital), were cultured in Dulbecco's Modified Eagle's Medium (DMEM) (Bioroc Pharmaceutical and Biotech Company LTD., Tianjin, China) with 10% heat-inactivated fetal bovine serum (FBS), 100 U/mL penicillin, and 100 mg/mL streptomycin in 10-cm dishes at 37°C in a humidified atmosphere of 5% CO_2_. A549 cells were grown in tissue culture plates (Corning USA) at a density of 4×10^5^/mL for 24 h before the explosion simulation. On the day of the explosion simulation, the culture plates were sealed with sterile, gas-permeable Mylar plate sealers (MP bio USA), and the edges of the plates and sealers were sealed with sterile tape before the explosion simulation.

### PFC-in-DMEM suspension

PFC was obtained from Shanghai Huajieshi Medical Company LTD, China. The molecular formula of the PFC used in our experiments was C_8_F_18_, which has a molecular weight of 438.06. At room temperature, the PFC had the following characteristics: vapor pressure of 60.9 mmHg; surface tension of 13.9 dynes/cm; capacity of oxygen delivery 52.1 mL O_2_/100 mL; boiling point pf 100–105°C; and density of 1.70–1.80 g/mL. PFC is not soluble, and we can not use it in cell culture. Therefore, we mixed PFC and DMEM at a ratio of 9:1 as previously described [15]. PFC and DMEM were mixed on ice at 21 kHz and 350 W in a transonic analogous ultrasonic unit for 10 sec. Then, the mixture was continuously shaken with a mini shaker (WuXiang Instrument and Meter Company. Ltd. Shanghai, China) to ensure that the mixture would not separate over time.

### Experimental report

A549 cells were divided into four groups as follows: (1) Blast group (B group), cells exposed to a 100-kPa blast; (2) Control group (C group), cells that did not receive any intervention; (3) PFC group (P group), cells incubated with PFC-DMEM suspension; and (4) Blast+PFC group (B+P group), cells exposed to a 100-kPa blast, and 10% PFC-DMEM suspension was added in a CO_2_ incubator.

### Morphological assay

Six hours (6 h) after blast simulation, cellular morphological changes were detected using a phase contrast microscope (Olympus, Japan). We fixed the cells with 4% paraformaldehyde in PBS. Then, the fixed cells were stained with β-tubulin and phalloidin. Next, cells were counterstained with 4’,6-diamidino-2-phenylindole (DAPI) for nuclear staining (shown in blue). The cells were observed using a confocal microscope (Olympus, Japan).

#### Apoptosis assay

The cells in different groups were digested and resuspended 6 h after blast simulation. Apoptotic cells were assessed using an Annexin V-fluorescein isothiocyanate (FITC)/propidium iodide (PI) apoptosis detection kit (Kengen biotech, China). We added 500 μL of Binding Buffer and 5 μL of Annexin V-FITC to the cells. Then, 5 μL of PI was added after 15 min of incubation. Finally, cells were analyzed by flow cytometry on a FACSCalibur (Becton-Dickinson, USA). All tests were performed in triplicate.

#### Enzyme-Linked Immunosorbent Assay (ELISA)

The cell supernatants were collected at different intervals (2, 4, 6, and 8 h) after blast simulation. IL-1β, IL-6 and TNF-α protein expression levels in the supernatants of the A549 cells were measured using ELISA kits (BD Bioscience, USA) according to the manufacturer’s protocol. Absorbance was measured at 450 nm with a microplate reader (Multiskan FC/ MK3, Finland). Three independent experiments were performed to collect and analyze the data. Each experiment was conducted with three replicate samples.

### Reverse Transcriptase-Polymerase Chain Reaction (RT-PCR)

At different time intervals after the blast (2, 4, 6, and 8 h), media were removed from each well, and cells were collected. Total RNA was extracted using TRIzol reagent (Takara, Japan), and reverse transcription to cDNA was performed using a reverse transcription system (Takara, Japan). A volume of 1 μL of cDNA was combined with primers and SYBR Green PCR Master Mix (Takara, Japan) to a final volume of 25 μL. The PCR conditions included 40 cycles of 95°C for 30 s, 60°C for 30 s, and 95°C for 5 min on a StepOnePlus^™^ Real-Time PCR System (BIO-RAD, USA). Threshold cycle (CT) values were determined by RT-PCR and normalized to the housekeeping gene β-actin. The relative expression levels of indicated genes were calculated using the 2^−ΔΔCt^ method. Primers are shown in Table 2.

**Table 2:** IL-1β, IL-6, TNF-α and β-actin primers for RT-PCR.

Name	Forward Primer	Reverse Primer
**IL-1β**	5’-GTACCTGTCCTGCGTGTTGA	5’-GGGAACTGGGCAGACTCAAA
**IL-6**	5’-CCAGAGCTGTGCAGATGAGT	5’-AGTTGTCATGTCCTGCAGCC
**TNF-α**	5’-TGAAAGCATGATCCGGGACG	5’-CAAAGTGCAGCAGGCAGAAG
**β-actin**	5’-CAAAGACCTGTACGCCAACACAGT	5’-ACTCCTGCTTGCTGATCCACATCT

### Measurement of Superoxide Dismutase(SOD) activity and Malondialdehyde (MDA) level

Treated cells and supernatants of cells were collected at different intervals (2, 4, 6, and 8 h) after blast simulation. We evaluated oxidative stress using the protocols of SOD and the MDA assay kits (Jiancheng Bioengineering Ltd., Nanjing, China). Protein content was measured according to the manufacturer’s instructions.

### Western blot method

Cells were collected at different time intervals after the blast (2, 4, 6, and 8 h). The protein content of each sample was determined with a bicinchoninic acid (BCA) protein assay kit (Applygen Technologies, Inc., Beijing, China). Cell lysates were denatured, and 40 μg of the protein was separated via 12% SDS–polyacrylamide gel electrophoresis. The protein was electro-transferred to polyvinylidene fluoride (PVDF) membranes for 2 h at 100 V. The membranes were blocked at room temperature with 5% skimmed milk in Tris-buffered saline (TBS) with 0.05% Tween-20 (TBS-T) for 2 h. After washing with TBS-T, membranes were incubated with the primary antibodies against NF-κB (1:1000 dilution, Cell Signaling Technology, USA), p-ERK (1:1000 dilution, Cell Signaling Technology, USA), p-JNK (1:1000 dilution, Cell Signaling Technology, USA), p-P38 (1:1000 dilution, Cell Signaling Technology, USA), Bax(1:10000 dilution, Cell Signaling Technology, USA), Bcl-2 (1:10000 dilution, Cell Signaling Technology, USA), Caspase-3 (1:10000 dilution, Cell Signaling Technology, USA), cleaved caspase-3 (1:10000 dilution, Cell Signaling Technology, USA) and β-actin (1:4000 dilution, Cell Signaling Technology, USA) overnight at 4°C. After washing, the membrane was incubated with goat anti-mouse IgG antibody (anti-rabbit or anti-mouse, 1:10000; Cell Signaling Technology, USA) at room temperature for 1 h. Protein bands were visualized by enhanced chemiluminescence (EMD Millipore, Billerica, MA, USA).

### Statistical analyses

Results are summarized as the means ± SD from three independent experiments. The statistical significance of differences was established by one-way ANOVA (analysis of variance) followed by Dunnett’s test. Two-tailed values of p<0.05 were considered statistically significant.

## Results

### Morphological changes of cells

**Fig 2:**
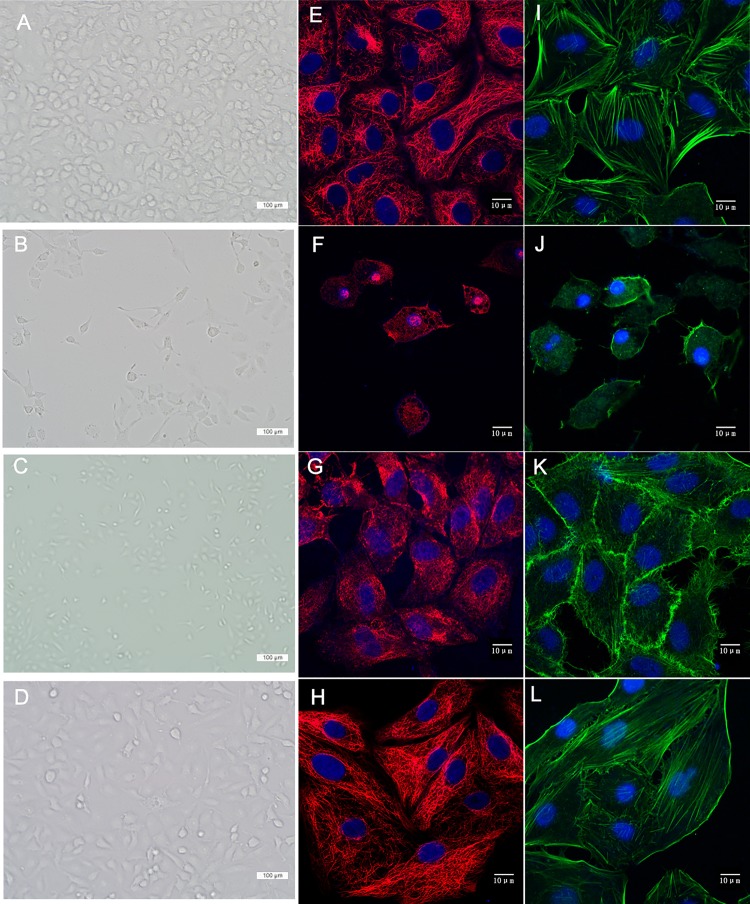
PFC inhibits blast–induced morphological changes in A549 cells. (A) (E) (I) Control group. (B) (F) (J) Blast group. (C) (G) (K) Blast +PFC group. (D) (H) (L)PFC group. (tubulin, red color; F-actin, green; and nuclei, blue). (Scale bars: 100 μm in (A, B, C, D) (scale bars: 10 μm in (D, E, F, G, H, I, J, K).

The morphological changes of A549 cells under light microscopy are shown in Fig 2A, 2B, 2C and 2D. A549 cells in the Control group were polygonal in shape and had intact cell-to-cell adhesions. The cell-to-cell adhesions were fewer in the Blast group than in the Control group. Cells in the Blast+PFC group maintained their polygonal shape, and were less damaged than the Blast group. For investigations of the changes in the micro-structure of A549 cells, we observed microtubules and microfilaments using laser confocal microscopy. Microtubules consist of polymerized α- and β-tubulin dimers, which are stained in red (β-tubulin) in Fig 2E, 2F, 2G and 2H. The microfilaments consist of polymers of actin, which are stained in green (phalloidin) in Fig 2I, 2J, 2K and 2L. Cells in the Control group had normal cuboidal morphology with a perinuclear cytoplasmic distribution of microfilaments and microtubules (Fig 2E and 2I). The nuclei were intact. The A549 cells in the blast group shrank, with microtubules and microfilaments grouped together. The cells in the Blast+PFC group were less damaged than the cells in the Blast group. These results indicated that PFC alleviates morphological changes induced by blast waves.

### PFC inhibits apoptosis in A549 cells

**Fig 3:**
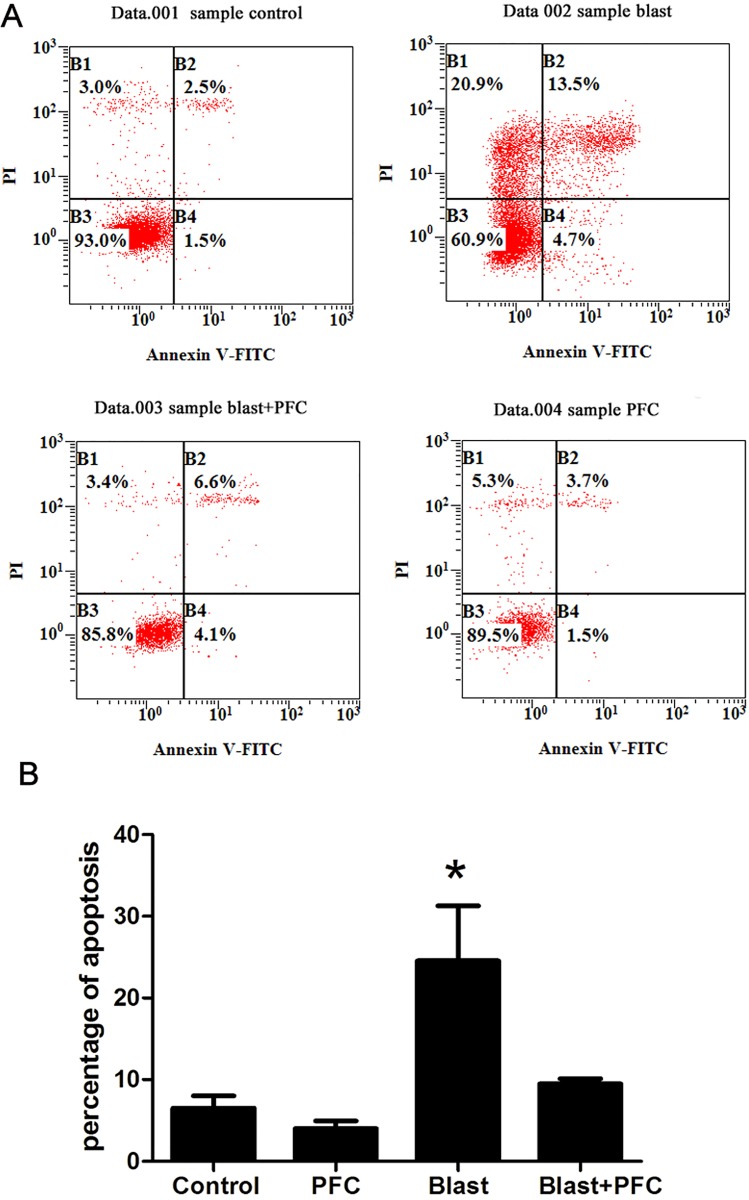
Effect of PFC on apoptosis in A549 cells. (A) After treatment with PFC for 6 h, apoptosis was observed using Annexin V/PI as previously described. (B) Analysis of the percentage of cells in apoptosis. Each bar represents the means of three independent experiments. * p<0.05 compared to the Control group, * p<0.05 compared to the Blast+PFC group.

**Fig 4:**
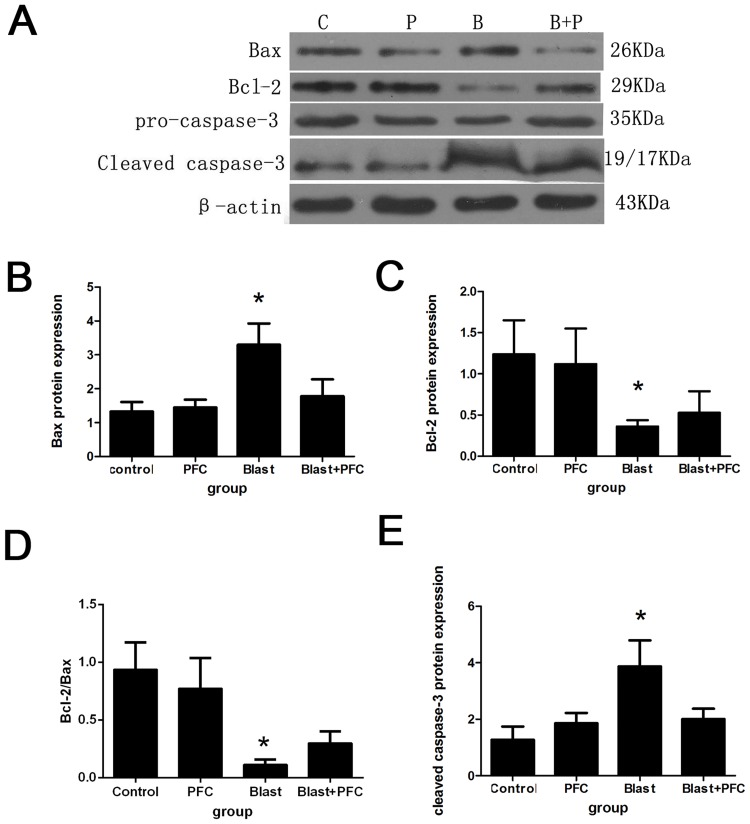
Effect of PFC on Bax, Bcl-2, and cleaved caspase-3 expression in A549 cells. (A) Western blot analysis was used on the protein lysates purified from A549 cells in different groups. β-actin was used as a control. (B) Densitometric analysis of Bax protein. (C) Densitometric analysis of Bcl-2 protein. (D) Analysis of the Bcl-2/Bax ratio. (E) Densitometric analysis of cleaved caspase-3 protein. * p<0.05 compared to the Control group, * p<0.05 compared to the Blast+PFC group.

The cells in different groups were collected 6 h after blast simulation. The number of apoptotic cells was quantified using Annexin V-FITC /PI. Flow cytometry was used to determine the effect of PFC on A549 apoptosis. The percentage of apoptotic cells was significantly reduced by PFC compared to the Control group (Fig 3). However, there was no significant difference in the percentage of apoptotic cells between the PFC and Control groups, indicating that PFC alone did not affect the apoptosis of A549 cells. To further investigate the potential mechanism, we determined the levels of proteins that regulate apoptosis (Bcl-2, Bax, and caspase-3) by Western blot. The results indicated that blast down-regulated the Bcl-2 and Bcl-2/Bax ratio, up-regulated Bax and blocked caspase-3 protein expression (Fig 4). PFC increased the Bcl-2 and Bcl-2/Bax ratio and decreased Bax and cleaved caspase-3 protein expression (Fig 4). We confirmed that PFC reduced A549 cell apoptosis induced by the blast wave by regulating Bcl-2, Bax and caspase-3 expression.

### PFC inhibits IL-1β, IL-6 and TNF-α secretion and mRNA expression

**Fig 5:**
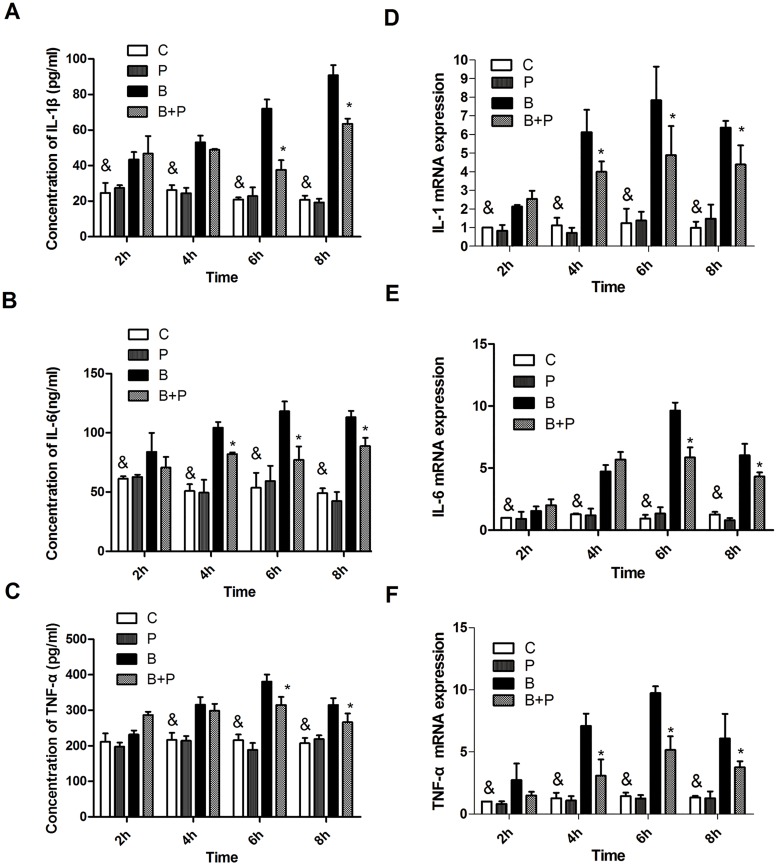
Protein and mRNA expression levels of IL-1β, IL-6, and TNF-α of A549 cells. (A) IL-1β, (B) IL-6 and, (C) TNF-α concentrations. (D) IL-1β, (E) IL-6, and (F) TNF-α mRNA expression. Results of three separate experiments are displayed in graphs and are expressed as the means ± SD. *p<0.05 compared to the Blast group, and & p<0.05 compared to the Blast group. **Note:** B: Blast group; B+P: Blast+PFC group; P: PFC group; C: Control group.

As shown in Fig 5A, 5B and 5C, 2 h after stimulation by the blast wave, IL-1β and IL-6 expression levels in the supernatant of A549 cells were significantly increased in the blast group compared to the Control group (p<0.05). At 4 h after blast, TNF-α expression was significantly increased in the blast group compared to the Control group. The Blast +PFC group showed significantly lower concentrations of IL-1β and TNF-α than the blast group at 6 and 8 h after PFC treatment. IL-6 expression in the Blast+PFC group was significantly lower than in the blast group at 4, 6 and 8 h after PFC treatment. IL-1β, IL-6 and TNF-α mRNA expression levels were similar as previously, but more sensitive than the protein in the supernatants. As shown in Fig 5D, 5E and 5F, the IL-1β, IL-6 and TNF-α mRNA expression levels began to increase at 2 h after blast, then reached a peak at 6 h after blast. The Blast+PFC group showed significantly lower IL-1β and TNF-α mRNA expression levels than the blast group at 4, 6 and 8 h after PFC treatment. IL-6 was suppressed by PFC at 6 and 8 h following treatment. These results indicate that PFC suppresses inflammatory cytokines induced by blast wave in A549 cells.

### Effect of PFC on SOD activity and MDA levelslevel in A549 cells exposed to blast waveswave

**Fig 6:**
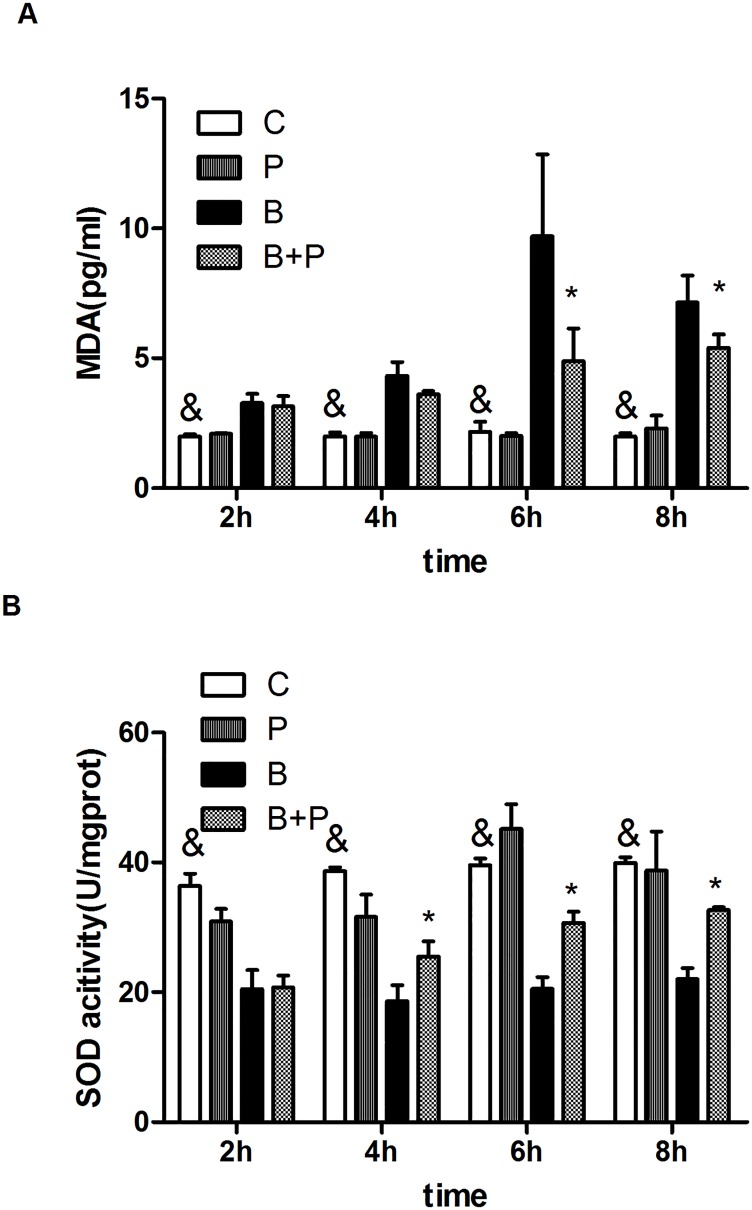
Effects of PFC on MDA and SOD in A549 cells exposed to blast waves. (A) MDA level. (B) SOD activity. Results of three separate experiments are displayed in graphs and are expressed as the means ± mean±SD. *p<0.05 compared to the Blast group, and & p<0.05 compared to the blast group. **Note:** B: Blast group; B+P: Blast+PFC group; P: PFC group; C: Control group.

To assess oxidative stress, SOD activity and the MDA level were evaluated in A549 cells. As shown in Fig 6A, the MDA levels were increased after exposure to the blast wave and were significantly attenuated 6h after treatment with PFC (p<0.05). The blast wave significantly decreased SOD activity levels in A549 cells compared to the Control group. The SOD activity was increased by treatment with PFC for 4h, 6h and 8h. (Fig 6B).

### Effects of PFC on NF-κB and phosphorylation of MAPK pathways

**Fig 7:**
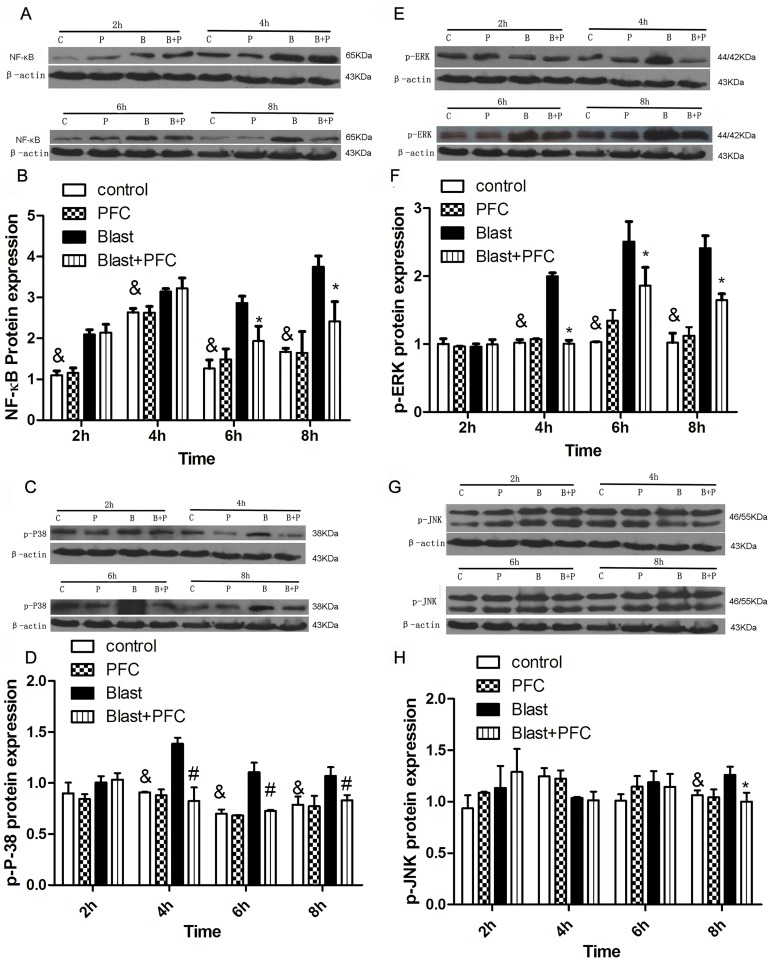
Effects of PFC on NF-κB p65, p-ERK, JNK, and p-P38 expression in A549 Cells. (A) Western blot analysis was used to assess NF-κB protein from lysates purified from A549 cells in different groups. β-actin was used as a control. (B) Densitometric analysis of blots is shown in A. (C) Western blot analysis was used to assess p-P38 protein in lysates purified from A549 cells in different groups. (D) Densitometric analysis of blots is shown in C. (E) Western blot analysis was used to assess the p-ERK protein in lysates purified from A549 cells in different groups. (F) Densitometric analysis of blots is shown in E. (G) Western blot analysis was used to assess the p-P38 protein in lysates purified from A549 cells in different groups. (H) Densitometric analysis of the blots is shown in G. Results were collected using the same method from three independent experiments; each was performed in triplicate. * p<0.05 vs blast group, and& p<0.05 vs blast group. **Note:** B: Blast group; B+P: Blast+PFC group; P: PFC group; C: Control group.

The NF-κB pathway is an important signaling pathway that regulates the expression of inflammatory mediators [22]. As shown in Fig 7A, 7B and 7C, NF-κB protein expression was significantly increased in the blast group compared to the Control group 2, 4, 6 and 8 h after blast. Treatment with PFC significantly reduced NF-κB protein expression at 6 and 8 h after PFC treatment. PFC significantly suppressed NF-κB expression compared to the group without PFC treatment in A549 cells. MAPK pathways, including ERK, p38 and JNK, modulate the production of inflammatory mediators and cytokines in A549 cells [23]. Therefore, we determined whether PFC suppressed MAPK activation in A549 cells. The levels of phosphorylated ERK, p38 and JNK were significantly increased by the blast wave, reaching a peak at 6 h after blast (Fig 7C, 7D, 7E, 7F, 7G and 7H). PFC inhibited ERK and p38 phosphorylation at 4, 6 and 8 h after blast compared to the group without PFC treatment (Fig 7C, 7D, 7E and 7F). PFC suppressed JNK phosphorylation at 8 h after blast (Fig 7G and 7H).

## Discussion

Our study showed that blast waves induced injury to human lung epithelial cells (A549) and that PFC reduced blast damage caused by apoptosis because it down-regulated the expression levels of Bcl-2/Bax and cleaved caspase-3 proteins. PFC also decreased IL-1β, IL-6 and TNF-α protein production and mRNA expression. Furthermore, we confirmed that PFC inhibited NF-κB activation and MAPK signaling, including ERK, JNK, and P38 phosphorylation. These findings help elucidate the potential mechanism by which PFC exhibits an anti-inflammatory effect against blast injury.

Blast wave induces cell injury and apoptosis in vivo. Perl M. exposed rats to blunt chest trauma using blast wave and found apoptosis of rat lung cells [24]. Seitz D.H. showed that apoptosis of type 2 epithelial cells of SD rat lung was caused by blast wave and was possibly related to the extrinsic death receptor [25]. In our experiment, we used Annexin V and PI staining to assess apoptosis of A549 cells and found that PFC decreased the apoptosis induced by blast waves in vitro. Therefore, we conclude that the anti-apoptosis effect of PFC is part of the mechanism by which PFC protects against blast-induced A549 cell injury. Previous studies reported similar results as ours. Qin et al found that vaporized PFC reduced intestinal mucosa apoptosis, possibly by inhibiting expression of the Bax gene and increasing expression of the Bcl-2 gene in rabbits [26]. Forgiarini L.A. confirmed that PFC decreased the expression of NF-κB, inducible nitric oxide synthase and caspase-3 in rats. Their expression levels were significantly lower than in another animal model of lung ischemia-reperfusion injury, suggesting a reduction in cell death [27]. Apoptosis was caused by a series of physiological and pathological signals. Through the regulation of death-related genes, death receptor pathways were activated, including Bcl-2, Bax and the caspase pathway [28,29]. For further investigation to elucidate the mechanism of action, we also analyzed Bax, Bcl-2, caspase-3 and cleaved caspase-3 protein expression by Western blot. Bcl-2 and Bax are key regulators of the cell apoptosis pathway. Bcl-2 family members change the mitochondrial membrane permeability and trigger the activation of caspases that lead to apoptosis [30]. PFC prevented cells from undergoing apoptosis by increasing the expression of anti-apoptotic Bcl-2 and decreasing the expression of pro-apoptotic Bax and caspase-3. Therefore, we concluded that increasing Bcl-2 expression and decreasing caspase-3 expression may be the possible mechanism for the anti-apoptosis effect of PFC. This report is the first time that PFC was shown to have an anti-apoptotic effect by regulating Bcl-2, Bax and caspase-3 expression in vitro.

The pathophysiological basis of lung blast injury is excessive alveolar inflammation with alveolar epithelial injury [28]. Accumulation of inflammation factors is a common mechanism of acute lung injury in vitro and in vivo [18,31]. Excessive inflammatory factor accumulation induces the depletion of major cellular components, such as lipids, proteins and DNA. Activation of the inflammatory reaction is the main reason for inflammation after blast injury. The inflammatory reaction is very important in the cure of lung injury. Previous animal experiments showed that severe internal and systemic inflammation caused by blast waves may lead to severe complications [25,31,32]. The overpressure from a blast can increase quantities of inflammatory factors, such as TNF-a, IL-6, and IL-1β in the plasma and bronchial alveolar lavage fluid [25,33]. This induced epithelial cell damage, leading to ARDS. Alveolar epithelial cells are often targeted by inflammatory and infectious agents, which participate in the initiation and progression of acute lung injury (ALI). Animal experiments have shown that lung blast injury induces alveolar epithelial cells to undergo apoptosis and increases the quantities of inflammatory factors, such as IL-1β [25]. The intensity of the inflammatory reaction indicates that cellular tissues injured by the blast wave receive therapy and restoration. We hypothesized that PFC may reduce inflammatory cytokines produced by alveolar epithelial cells. To verify this hypothesis, we exposed A549 cells to blast overpressure and then treated them with PFC and then measured the quantities of inflammatory cytokines. The levels of TNF-a, IL-1β, and IL-6 were significantly increased, thus verifying our hypothesis. That ROS accumulation plays an important role in the pathogenesis of ALI [34–36]. MDA is the final product of peroxidation, and SOD is critical protective antioxidants that protect the epithelium and endothelium of the lung from oxidant injury and inflammation. Our results demonstrate that blast injury increases MDA and decreases SOD activity levels. PFC treatment alleviates oxidative stress, which partially contributes to protection from blast injury. The results of our experiments are consistent with those of previous studies. Rotta AT et al found that perflubron attenuates oxidative damage induced by linoleic acid in rat pulmonary artery endothelial cells [37].

To further investigate the mechanism of action, we determined NF-κB and MAPK protein expression levels. NF-κB and MAPK are important transcription factors that bind to DNA regulatory sequences in cells and control the rate of gene expression. The experiments indicated that PFC down-regulated the synthesis and secretion of cytokines and chemokines through NF-κB and ERK, JNK, and P38, similar to the anti-inflammatory mechanism of PFC on A549 cells. These results are consistent with the conclusion that PFC inhibits NF-κB activity to protect against LPS-induced inflammatory injury [38]. To our knowledge, this is the first experiment showing that PFC inhibits inflammatory damage and apoptosis induced by blast wave via inactivation of the NF-κB and MAPK signaling pathways.

However, there are limitations to our study. First, lung blast injury is generally classified into four types [39]. Primary blast injury mainly affects air-filled organs, such as the ears, lungs, and gastrointestinal tract [40]. The mechanism of primary lung blast injury includes three possible modes: spallation, inertia, and implosions. The cell injury model in vitro simulated with a shock tube cannot completely mimic actual lung blast injury; therefore, further studies are needed to investigate the effect of PFC on lung blast injury in vivo.

**Fig 8:**
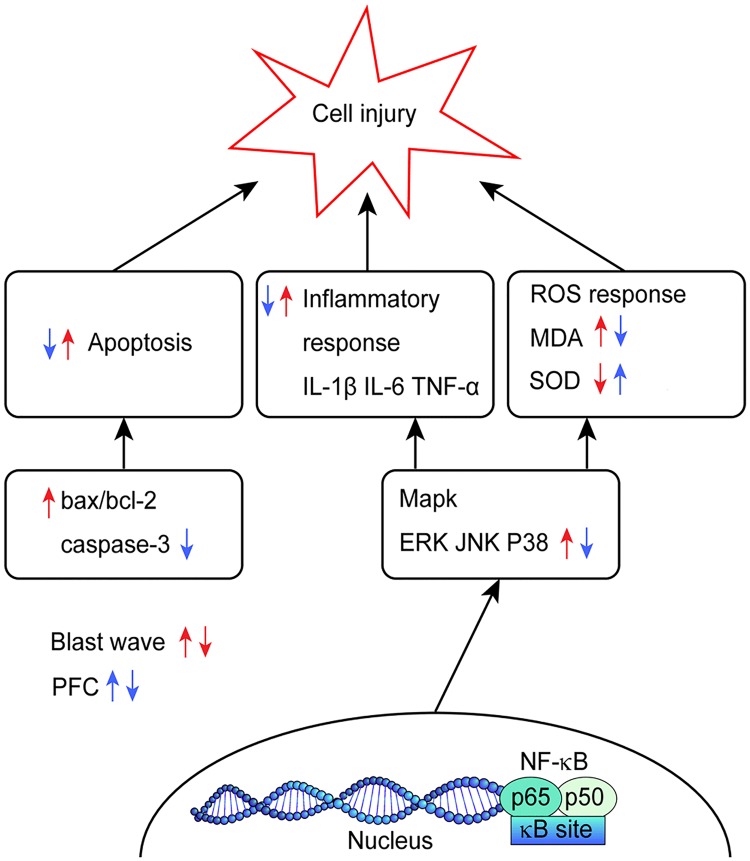
Schematic outlining the possible mechanism of PFC inhibition of blast-induced A549 cell injury.

In brief, we found that PFC reduced cell injury induced by blast wave in vitro. The possible mechanism of the action of PFC in inhibiting blast-induced injury is shown in Fig 8. Inhibition of Bcl-2/Bax, caspase-3, and NF-κB activation and regulation of MAPK signaling may be the possible mechanisms for the effects of PFC. For therapeutic strategies, these reagents used for anti-inflammatory and anti-oxidative stress research provide support for further therapeutic approaches targeting the mechanism of action of PFC in lung blast injury, which is not yet completely clear. PFC facilitates A549 cell recovery from blast damage. This protection may be more useful in situations of severe blast lung injuries with ALI/ARDS.

## Supporting information

S1 File Effect of PFC on apoptosis in A549 cells. (RAR)

S2 File Effect of PFC on Bax, Bcl-2, and cleaved caspase-3 expression in A549 cells. (RAR)

S3 File Protein and mRNA expression levels of IL-1β, IL-6, and TNF-α of A549 cells. (RAR)

S4 File Effects of PFC on MDA and SOD in A549 cells exposed to blast waves. (RAR)

S5 File Effects of PFC on NF-κB p65, p-ERK, JNK, and p-P38 expression in A549 cells. (RAR)
